# The usage of population and disease registries as pre-screening tools for clinical trials, a systematic review

**DOI:** 10.1186/s13643-024-02533-0

**Published:** 2024-04-23

**Authors:** Juliette Foucher, Louisa Azizi, Linn Öijerstedt, Ulf Kläppe, Caroline Ingre

**Affiliations:** 1 Department of Clinical Neuroscience, Karolinska Institutet, Stockholm, Sweden; 2 Department of Neurology, Karolinska University Hospital, Stockholm, Sweden

**Keywords:** Clinical trials, Registry, Pre-screening, Patient selection, Review

## Abstract

**Objective:**

This systematic review aims to outline the use of population and disease registries for clinical trial pre-screening.

**Materials and methods:**

The search was conducted in the time period of January 2014 to December 2022 in three databases: MEDLINE, Embase, and Web of Science Core Collection. References were screened using the Rayyan software, firstly based on titles and abstracts only, and secondly through full text review. Quality of the included studies was assessed using the List of Included Studies and quality Assurance in Review tool, enabling inclusion of publications of only moderate to high quality.

**Results:**

The search originally identified 1430 citations, but only 24 studies were included, reporting the use of population and/or disease registries for trial pre-screening. Nine disease domains were represented, with 54% of studies using registries based in the USA, and 62.5% of the studies using national registries. Half of the studies reported usage for drug trials, and over 478,679 patients were identified through registries in this review. Main advantages of the pre-screening methodology were reduced financial burden and time reduction.

**Discussion and conclusion:**

The use of registries for trial pre-screening increases reproducibility of the pre-screening process across trials and sites, allowing for implementation and improvement of a quality assurance process. Pre-screening strategies seem under-reported, and we encourage more trials to use and describe their pre-screening processes, as there is a need for standardized methodological guidelines.

**Supplementary Information:**

The online version contains supplementary material available at 10.1186/s13643-024-02533-0.

## Introduction

Clinical trials are essential in allowing the scientific transition from basic research to clinical practice, whether the trials are about drug development or other types of non-drug interventions [1]. Clinical trial protocols are the trial documents of reference, detailing every step for participants enrolled in the trial. One of the critical protocol sections, is the list of eligibility criteria [2]—if this list is clinical trial specific, it will often include recurring criteria within the same field or disease area, with trial specific cut-off differences [2]. For example, when conducting a trial for the neurodegenerative disease amyotrophic lateral sclerosis (ALS), it will in the majority of cases include vital capacity (VC) measurement as a trial eligibility criteria. Some ALS trials only include patients with a VC above 50% (NCT05633459), while others ask for patients with VC equal or superior to 65% (national clinical trial NCT, NCT04248465). Enrolling the right participants in a clinical trial is essential as it (1) could allow for a personalized medicine approach [3–6], (2) might be the only option to access drugs in development for patients suffering from diseases with no cures [7–10], (3) should ensure that motivated participants complete the entire study without dropping out, and thus ultimately maximize patient retention [11–14], and (4) in the end ensures good quality clinical trial data [15]. However, trial enrollment can also be challenging, especially to efficiently identify the above mentioned potentially eligible candidates during the pre-screening process [16–18]. This requires the identification of participants meeting the most stringent criteria and ultimately highly likely to be successful during the screening process. The pre-screening procedure is crucial as it decreases the screen failure rate, which drastically varies between trials across disease areas and countries [19–22]. Considering that screen failures are associated with participant burden while also negatively impacting the study budget, there is a need to develop clinical trial recruitment strategies targeting these aspects [23–26].

Typically, trial pre-screening is staff-bound with a designated staff member in charge of the pre-screening process. Research teams usually have team specific pre-screening processes, as there are no national consensus, guidelines nor universal standard operating procedures (SOPs) on how to conduct the pre-screening for clinical trials. A typical pre-screening process may include an internal check of the hospital medical journals in paper format, a review of electronic medical journals, a review of medical journals sent via traditional mail in case of a referral, direct emails from patients emailing a research team, and more [27–30]. This creates a pool of information derived from several sources, with no standardized system assuring quality and replicability, not allowing an audit trail for quality assurance and control, and overall creating inequitable trial access for patients [31]. Clinical trial eligibility criteria will often include both demographic data and disease specific information. This information is captured in most disease registries/population registries/patient registries, and there is an growing interest in using such registries for pre-screening due to the high quality and easy accessible data [32–35]. Such disease registries/population registries/patient registries are to be distinguished from other types of databases such as electronic health records (EHR). For the purpose of this review, we will use the term patient registry when referring to a specific database aiming to capture data on all patients from a patient population in a specific site, state, or country [32, 36, 37].

The usage of population and disease registries for trial pre-screening has previously been investigated through a literature review of the period 2004–2013, reporting limited registry use for clinical trial pre-screening, but advocating for a more systematic usage as this was deemed an efficient method [38]. The combined use of registries and medical record data has been described as optimizing trial recruitment [23], and we have since 2013 observed an explosion of clinical trials in many different fields as reported by the International Clinical Trial Registry Platform (ICTRP) of the World Health Organization (WHO) [39]. The ICTRP collects trial registration information from different databases such as Clinicaltrial.gov and reported 34,291 clinical trials in 2013 versus 59,964 clinical trials in 2021. The increasing number of clinical trials globally highlights the need for efficient and equitable pre-screening processes, but also for an updated review considering the last review on this topic was not conducted with a systematic methodology [38].

In this systematic review, we characterized the use of population and disease registries as a pre-screening tool for clinical trials not discriminating between drug and non-drug trials. We included publications published between January 2014 and December 2022, as a non-systematic review covered the 2004 to 2013 timeframe. We aimed to describe the type of registries used, disease areas, type of clinical trials linked to the registry-based pre-screening, and potential assets the method brought to the pre-screening process.

## Methods

### Inclusion and exclusion criteria

Citations and references obtained from the search were screened using the Rayyan software and our set of inclusion and exclusion criteria are listed in Table 1. Eligible studies had to be in English, from peer-reviewed journals, reporting the use of population/patient/disease registries for trial pre-screening. Included studies also needed to be set in trials on patients and not on healthy individuals. Studies had to have been published in our targeted window between January 2014 and December 2022, and abstracts had to be available for review. Finally, we included studies of high to moderate quality, as evaluated through the List of Included Studies and quality Assurance in Review (LISA-R) tool. Since there was no standardized tool to judge the quality of the included studies, we developed a quality assurance tool, the LISA-R. This quality assurance checklist was developed using guidance provided on the Parsifal platform for systematic reviews, a platform providing support for researchers conducting reviews and wishing to establish new quality assurance tools. The tool consists of 11 items in which each item was judged on a two-level scale (yes/no) (LISA-R blank tool available in Supplementary material 2). For each “yes”, one point was attributed, giving a scale range from 0 to 11. An overall score > 8 was interpreted as high quality, 6–8 moderate quality, and < 6 low quality.

**Table 1:** Inclusion and exclusion criteria used for article selection in Rayyan

1	**Inclusion**	**Exclusion**
2	Articles in English (papers and/or abstracts)	Articles in another language than English (papers and/or abstracts)
3	Peer-reviewed	Registry use not fitting our scope: use of recruitment registry, no use of registries, or use of a registry for other reason than patient pre-screening and selection
4	Use of population registry for trial pre-screening	Prevention clinical trials with healthy individuals
5	Use of patient registry for trial pre-screening	Sole use of healthcare database
6	Use of disease registry for trial pre-screening	Gray literature
7	Clinical trials for patients (not healthy individuals)	Published outside our window 2014–2022
8	Published within our window 2014–2022	Not a clinical trial, or for a trial not yet conducted
9	Abstract available for review	No abstract available
10	High to moderate quality studies, as evaluated through LISA-R	Low quality studies, as evaluated through LISA-R (for phase 3 of study selection)

### Search and selection strategy

The study protocol was registered in PROSPERO with the identification number CRD42023433968 and followed the PRISMA requirements [40]. A literature search was performed in the following databases: MEDLINE, Embase, and Web of Science Core Collection. The last search was conducted on June 22, 2023.

The search strategy was developed in MEDLINE (Ovid) in collaboration with librarians at the Karolinska Institutet University Library. For each search concept, medical subject headings (MeSH-terms) and free text terms were identified (Supplementary material 1). The search was then translated, with Polyglot Search Translator used for the translation of the controlled vocabulary [41], into the other databases.

Language restriction was made to English and the search was limited to years 2014–2022 as a previous non-systematic review covered the 2004–2013 period [38]. De-duplication was done using the method described by Bramer et al. [1]. One final step was added to compare digital object identifiers to finalize de-duplication. The full search strategies for all databases are available in supplementary material (Supplementary material 1). The review of papers was conducted by two of the authors (JF and LA) independently and then cross-checked. A third author (CI) was asked to solve selection conflicts if they arose, by setting-up a meeting where JF and LA could expose their process and CI could make the final decision. A first review process (phase 1) was done based on titles and abstracts only, while the second review was of full texts (phase 2). Only the publications of moderate and high quality as per the LISA-R tool were included in the final search (phase 3).

### Data extraction

Data extraction was conducted by two of the authors (JF and LA) reading the full texts and summarizing information in table format through an excel form. This data extraction form was created for the sole purpose of this systematic review. The extraction form included the information we wished to extract from the included studies: trial type (drug trial versus non-drug trial), clinical trial name, NCT number, registry name and scope, patient population, and age. In order to specifically look into enrollment and pre-screening rates, we extracted the number of patients identified through the registries, number of patients eligible for the trial in question, and number of patients enrolled in the clinical trial. Different enrollment rates were calculated when possible and represented by percentages: (1) comparing the number of patient enrolled to the number identified in the pre-screening process and (2) comparing the number of patient enrolled to number of patients actually eligible after screening.

## Results

### Review process

**Fig. 1:**
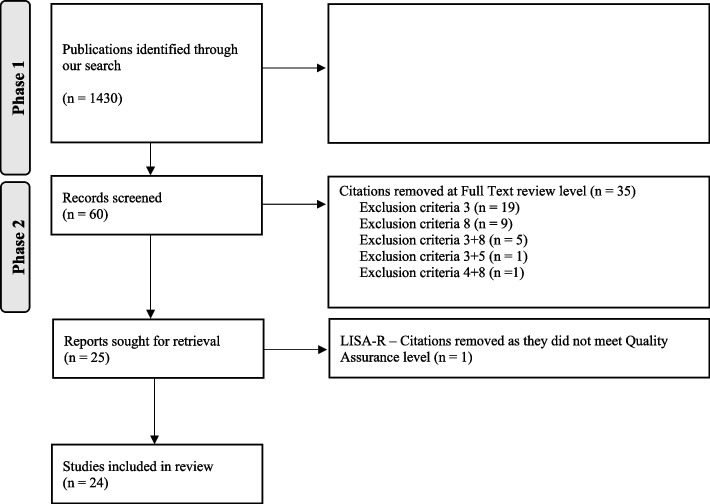
Flowchart of the selection process

A total of 1430 citations were identified through the literature search. Out of them, 1369 were excluded based on titles and abstract review as they did not meet inclusion criteria (Table 1). One citation was excluded as a duplicate (Fig. 1). The 60 remaining publications were reviewed by reading the full text and 35 publications were subsequently excluded as they did not meet inclusion criteria. The remaining 25 publications were assessed using the LISA-R tool and the articles of low quality were excluded, ending up with 24 included papers (Supplementary material 3a and b). The list of excluded papers is available upon request.

### Included articles—descriptive characteristics

Out of the 24 articles included and reporting the use of a population/disease registry for a clinical trial pre-screening [42–65], we identified nine disease domains/fields: nine articles were associated with oncology, three articles with the cardiovascular field, two papers with inflammatory diseases, one paper with autoimmune diseases, one paper with pulmonary diseases, one paper with hepatology, two papers with endocrinology, four papers with neurological and/or neuromuscular disorders, and one paper associated with metabolic disorders. Among the included articles, a majority were based on registries from the USA (13 out of 24) [53–65], with the rest describing either single-European-country registries or other international registries, and one registry from Israel [42–52]. Descriptive characteristics of the included papers are available in Supplementary material 4.

### Included articles—trial/registry and patient population’s characteristics

In terms of registry scope, 15 of the 24 included studies reported using national registries for trial pre-screening (Supplementary material 4, papers 12/18/20/16/10/9/13/21/24/14/4/8/5/6/23) (respectively [42, 43, 45, 46, 48–51, 54, 57–59, 62, 63, 65]), and 9 of 24 studies reported using local registries (Supplementary material 4, papers 22/17/24/1/19/7/2/5/3) (respectively [44, 53–56, 60–62, 64]). Local registries were either at a state level (Supplementary material 4, papers 17 and 19) [53, 56], or a specific site level (Supplementary material 4, papers 22/1/7/2/3) (respectively [44, 55, 60, 61, 64]). Two included studies reported using a combination of local and national registries (Supplementary material 4, papers 24 and 5) [54, 62]. Only two international registries were reported being used for trial pre-screening (Supplementary material 4, papers 15 and 11) [47, 52]. We observed that 50% of the included studies reported pre-screening for drug trials (Supplementary material 4, papers 22/16/15/10/21/1/14/8/7/2/6/23) (respectively [44, 46–48, 51, 55, 57, 59–61, 63, 65]), and 50% of non-drug trials (Supplementary material 4, papers 12/18/20/9//13/11/17/24/19/4/5/3) (respectively [42, 43, 45, 49, 50, 52–54, 56, 58, 62, 64]). Only one registry and trial targeted a pediatric population (Supplementary material 4, paper 14) [57]. In total, we estimate that over 478,679 patients were identified through registries in this review. However, three studies did not report any patient numbers [59, 61, 62]. The number of patients eligible in the individual studies varied between 59 and 16,091 [43, 45].

Characteristics linked to the clinical trials, the registries, and the patient population included in this systematic review are available in Table 2.

**Table 2:** Included papers, clinical trial/registry/patient population characteristics

**#**	**Trial type**	**Clinical trial name**	**NCT number**	**Registry**	**Registry scope**	**Patient population**	**Age**	**Trial enrollment out of registry pre-screening**				
								**Patients identified through the registry**	**Patients eligible for the clinical trial**	**Patients enrolled in the clinical trial**	**Enrollment rate compared to patients identified**	**Enrollment rate compared to patients eligible**
**1**	Drug trial	General eligibility for lung cancer trials and studies	N/A	Cancer registry	Local (center specific)	Patients with lung cancer	Not specified	1	N/A	N/A	N/A	N/A
**2**	Drug trial	General eligibility for cancer trials	N/A	Cancer registry	Local (center specific)	Patients with cancer	≥ 30	N/A	N/A	N/A	N/A	N/A
**3**	Non-drug trial	IMPACT	NCT03569605	UNC Health Registry/Cancer Survivorship Cohort	Local (center specific)	Young adult cancer survivors (YACS)	18–39	101	N/A	N/A	N/A	3.60%
**4**	Non-drug trial	CONTROL-RA	N/A	ArthritisPower patient registry	National	Patients with rheumatoid arthritis	≥ 19	27,000	1254	234	0.87%	18.60%
**5**	Non-drug trial	EXERT	NCT02814526	National and local registries	Both national and local registries	Patients with mild cognitive impairment	65–89	N/A	N/A	N/A	N/A	N/A
**6**	Drug trial	16 trials	N/A	National ALS Registry	National	Patients with amyotrophic lateral sclerosis	≥ 18	7030	N/A	2000	28.40%	N/A
**7**	Drug trial	General eligibility for “for all available pancreatic cancer clinical trials”	N/A	Abramson Cancer Center cancer registry	Local (center specific)	Patients with pancreatic cancer	≥ 18	233	142	N/A	N/A	N/A
**8**	Drug trial	General eligibility for cancer trials	N/A	Duke Molecular Registry of Tumors	National	Patients with cancer	≥ 18	N/A	N/A	32	N/A	N/A
**9**	Non-drug trial	Effect evaluation of Oncokompas 2.0	N/A	Netherlands Cancer Registry	National	Patients with cancer	≥ 18	2953	N/A	625	21.20%	N/A
**10**	Drug trial	SHERBOC	NCT03241810	PRAEGNANT registry	National	Postmenopausal women with heregulin positive, hormone receptor positive, HER2 negative metastatic, unresectable breast cancer	≥ 18	2769	125	N/A	N/A	N/A
**11**	Non-drug trial	TARGET-AC	NCT02520180	All comer disease registry	International	Patients with coronary artery disease	≥ 18	912	789	131	14.40%	16.60%
**12**	Non-drug trial	TASTE	NCT01093404	Swedish Coronary Angiography and Angioplasty Registry (SCAAR)	National	Patients with a diagnosis of ST-segment elevation myocardial infarction	≥ 18	11,956	7259	7244	60%	99%
**13**	Non-drug trial	DYVINE	NCT01225614	DM-Scope registry	National	Patients with myotonic dystrophy	≥ 18	2648	N/A	N/A	N/A	N/A
**14**	Drug trial	Generic eligibility for “current” pediatric type 2 diabetes	N/A	Pediatric Diabetes Consortium T2D Registry	National	Pediatric patients with diabetes	10 to < 18 years	956	604	N/A	N/A	N/A
**15**	Drug trial	COMPASS	NCT01776424	Reduction of Atherothrombosis for Continued Health (REACH) registry	International	Coronary artery disease Patients with coronary artery disease or peripheral artery disease	≥ 18	65,531	16,875	9126	14%	54%
**16**	Drug trial	6 trials	N/A	UK Primary Sjögren’s Syndrome Registry	National	Patients with Sjögren’s syndrome	Not specified	688	681	N/A	N/A	N/A
**17**	Non-drug trial	COURAGE	NCT02250053	Pennsylvania Cancer Registry	Local (Pennsylvania)	Patients who survived colon cancer	≥ 18	1435	1433	39	2.70%	2.70%
**18**	Non-drug trial	ASCEND	NCT00135226	Patient registry	National	Patients with diabetes	≥ 40	300,188	16,091	9013	3%	56%
**19**	Non-drug trial	COPD-SMART	NCT01108991	Patient registry	Local (Texas)	Patients with COPD	≥ 45	1666	924	325	19%	35%
**20**	Non-drug trial	DILT1D	NCT01827735	Patient registry	National	Patients with typ 1 Diabetes	18–50	477	59	10	2%	17%
**21**	Drug trial	OPTEX	NCT00803309	Patient registry	National	Patients with chronic HCV	≥ 18	1006	226	104	10%	46%
**22**	Drug trial	Effect of Alfacalcidol on multiple sclerosis-related fatigue	N/A	Patient registry	Local (center specific)	Patients with MS and fatigue	18–55	600	259	158	26%	61%
**23**	Drug trial	VERVE	NCT02538341	Consortium of Rheumatology Researchers of North America (CORRONA) disease registry	National	Patients with rheumatoid arthritis	≥ 50	50,000	12,000	Not indicated	N/A	N/A
**24**	Non-drug trial	Un-named trial	N/A	California Cancer Registry	Local and national	African American or Latino breast cancer survivors	≥ 18	529	252	221	42%	48%

### Included articles—patient trial enrollment out of registry pre-screening

Seven of the 24 papers included reported mock enrollment numbers as they were from retrospective studies, followed by a simulated enrollment performance out of registry usage for pre-screening [43, 45–48, 56, 65]. Only 11 studies reported the full pre-screening process from patient identification to eligibility evaluation and finally trial enrollment, with highly heterogenous numbers and enrollment rates [42–45, 47, 51–54, 56, 58] (Table 2).

In the 11 papers that fully reported numbers from identified, eligible, and enrolled patients (Supplementary material 4, papers 12/18/22/20/15/21/11/17/24/19/4) (respectively [42–45, 47, 51–54, 56, 58]), we observed different enrollment rates compared to patients first identified through their respective registries with a span of 0.87% [58] to 60% [42]. When calculating the enrollment rates compared to patients deemed eligible from their respective registries, results went to a span of 18.6% [58] to 99% [42].

### Quality assessment and risk of bias

In order to assess quality of the included paper and their risk of bias (RoB), we developed a quality checklist available as Supplementary Table 2. Over the 24 studies describing registry use for clinical trial pre-screening, the majority of the 11 quality questions selected in our checklist were met (Supplementary material 4, papers 12/18/22/20/16/15/9/21/11/17/24/1/19/14/4/8/7/2/5/6/3/23) (respectively [42–47, 49, 51–65]), with only 2 studies checking for all items (Supplementary material 4, papers 10 and 13) [48, 50].

In 11 of 24 studies, NCT numbers were not mentioned (Supplementary material 4, papers 22/16/9/24/1/14/4/8/7/2/6) (respectively [44, 46, 49, 54, 55, 57–61, 63]) even though trial names were documented in ten of them (Supplementary material 4, papers 22/16/9/1/14/4/8/7/2/6) (respectively [44, 46, 49, 55, 57–61, 63]). Conflict of interests were disclosed in the vast majority of the studies with only one paper not disclosing them (Supplementary material 4, paper 19) [56].

In terms of pre-screening methodology, only six studies (Supplementary material 4, papers 12/22/16/9/21/11) did not specify in what way the registry was used to perform trial pre-screening (respectively [42, 44, 46, 49, 51, 52]).

Overall, with a majority of quality marks being met using the LISA-R tool, we estimate the quality of the studies included in this review from moderate to high, as the majority of them provide enough information to replicate their methods and findings in using a population registry for a clinical trial pre-screening.

## Discussion

We conducted a systematic review including 24 studies reporting the usage of population and disease registries for clinical trial pre-screening between January 2014 and December 2022. We aimed to describe the type of registries used, disease areas, type of clinical trials linked to the registry-based pre-screening, and potential assets the method brought to the pre-screening process. Our study shows that the use of registries for clinical trial pre-screening is very diverse in terms of registry type (international, national, local and statewide, local and site specific). We observed less diversity in terms of geography since a majority of the studies included in the review were using registries from the USA, with only one study using Nordic registry data. The US dominance is surprising knowing that for example the Nordic countries have been extensively described for their use of registries and registry-research [66–73]. This could possibly be explained by “recruitment registries” or “research ready cohorts” are being developed and were excluded from our review (Table 1) as they either include healthy participants who are at risk of developing diseases in the future or patients who are solely in registries due to their interest in participating in clinical trials [38, 74–79]. Our search highlighted that such recruitment registries seem to be extensively used in Alzheimer and dementia research [80–87]. This could explain why patient registries may surprisingly not be the first in line of use for trial pre-screening, as “recruitment registries” are blooming to support different trials. However, recruitment registries should be carefully considered as they bring ethical concerns. Indeed, they can lead to consenting patients already enrolled in other trials, or having to deal with changes in patient’s disease status not being updated [88].

In terms of disease areas, 11 of the 24 included studies reported use in either cardiovascular health or oncology. This is aligned with the ICTRP website that reports oncology and cardiovascular trials at the 1^st^ and 3^rd^ position for the numbers of trials by health category (the 2^nd^ place being for neuropsychiatric conditions) [39]. We found half of drug trials (compared to other interventions) in our included studies with 12 publications of the 24 included reporting registry usage for drug trial pre-screening [44, 46–48, 51, 55, 57, 59–61, 63, 65]. This reflects the importance of drug trials in the clinical trial landscape, but should also be considered in relation to the disease domains they are associated with, as certain diseases call more for drug trials than non-drug trials.

The main challenge of this systematic review was that it did not follow the traditional Patient/Exposure/Comparator/Outcome (PECO), or Patient/Intervention/Comparator/Outcome (PICO) as usually recommended [89]. The reason was that such traditional method did not fit our study purpose. If a PECO was to be outlined then our patient group could be people with diseases (as we are investigating patient registries/disease registries/population registries). The exposure could be to be included in a registry, with the consequence that the inclusion of all exposures indeed would not allow for intervention-specific conclusion. However, it does increase the generalizability of our results as we included registries from different disease areas. In this study, we could not define a control group or comparator group, since all included studies used different set-ups. For that reason, we did not specify the comparator group in our search strategy, and we included all control groups in our analyses. This could possibly have led to a dilution of the results, but we believe that it extended the external validity of our study. Regarding the outcome of the PECO, it could be the number of patients identified/eligible/enrolled for a clinical trial. However, here we aimed to describe the landscape of the usage of registries for clinical trials via a systematic review approach, meaning the final enrollment numbers were not an indicator of success or failure.

We observed a large variation in enrollments rates compared to patients deemed eligible from their respective registries. Higher and lower rates should not be interpreted as successes or failures, as these are directly linked to patient population, registry types, and moreover trial inclusion and exclusion criteria. One may identify a great number of patients in a registry but have very restrictive inclusion and exclusion criteria that will only make a fraction of your identified patients eligible, which should not be interpreted as a default in methodology. Similarly, only a fraction of the eligible patients will be enrolled due to various reasons: trial may be linked to a high patient burden leading to only a few patients consenting it, patients may live far away from the trial center and do not wish to travel, or patient may decline research participation for other reasons. However, these numbers should be considered when discussing pre-screening and recruitment methodology with respect of their specific constraints and challenges. This could allow study teams and field experts to better understand their recruitment workflow and the parameters influencing these rates.

Our second challenge was that available checklists of RoB tools did not fit our research question, leading us to develop our own quality assessment checklist of 11 items, the LISA-R tool (Supplementary material 2). This may be seen as a limitation as this did not allow us to produce a RoB score. However, the checklist allowed us to obtain a rigorous and traceable quality assessment tool. Through this checklist, we estimated that all included studies were either of high or moderate quality. If the checklist was developed using guidance provided on the Parsifal platform for systematic reviews, it was not tested prior to this review as it was specifically designed for this study, and is the pilot try of the LISA-R [90]. In the future, we aim like to validate this tool in a larger and dedicated study.

Thirdly, we only included studies published in English, and we do acknowledge that more studies fitting in our scope may have been published in other languages. However, as English is the main scientific language, we would not expect the additional studies to change our observations and conclusions.

Lastly, we need to acknowledge the risk of bias in registry inclusion. We know that certain registries may be highly effective at capturing patients, like the Swedish Motor Neuron Disease (MND) National Quality Registry including 99% of MND patients in the Stockholm Region [91]. However, this is not the case for all registries and varies geographically. For an optimal and efficient national registry based pre-screening, one would need to have 100% of a national patient population entered in the registry in order to be truly representative. We observed that five of the included studies reported using a local registry that was at center scale (Supplementary material 4, papers 22/1/7/2/3) (respectively [44, 55, 60, 61, 64]), for which we would assume a 100% adherence between patient followed up at the site and the site registry. However, this would depend on site resources and site staff’s adherence to registering patients into the site’s registry. A recent Cochrane review on “*strategies to improve recruitment to randomized trials*” only mentioned two papers reporting pre-screening methods, both judged with high risk of bias and therefore not included in the final analyses, highlighting the blind spot surrounding trial pre-screening methodology [92–94].

Of the 24 included studies in our review, 14 reported benefits for using population registries in the trial pre-screening process [42, 43, 45, 48, 50, 55, 56, 58–61, 63–65]. Advantages of this methodology has been described as cost-efficient trial recruitment and benefits patients in countries with small populations or low population density in specific areas and also patients with rare diseases [55, 61, 65, 95, 96]. Ethically, using a population or disease registry for clinical trial pre-screening in a systematic manner would guarantee for all patients to be considered in the same equitable way, not discriminating between patients clinically followed up in large university hospital and patients living in remote areas. However, in order to be representative of the full disease population one would need to make sure 100% of patients are enrolled in their disease registries in order for it to be absolutely representative. Using registries only capturing a small portion of the patient population is introducing a potential risk of bias, and therefore efforts to capture all patients in disease registries should be maintained. Furthermore, the use of registries for pre-screening patients for clinical trials also gives an opportunity for a trial in a real-world setting and increases the evidence value of the trial [55, 61, 65, 95, 96].

Furthermore, using registries for trial pre-screening increases the reproducibility of the pre-screening process across trials, increases the chances of all registry patients to be considered, and allows for implementation and improvement of quality assurance processes. As most studies reported use of national registries, this highlights the consideration of a patient population on a national level, maximizing efficiency and representativity of the pre-screening process. However, to apply such methodology, it is essential that the consent forms include information about the data might be used to confirm clinical trial eligibility and that trials may be offered to the included patients [97]. One might argue that these benefits are not reflecting the current reality: since January 2014 there was a mean of 50,000 clinical trials running each year [39] and only 24 studies between 2014 and 2022 reported using population registries for pre-screening despite advantages with this method. However, the literature is known to under-report recruitment strategies in clinical trials, from protocols to publications [98, 99]. This leads to restrictive data, as this systematic review only reflects research that reported registry use in a clinical trial pre-screening setting. It is important to consider more clinical trials may pre-screen and recruit patients from registries without reporting it neither in their protocol nor in their published methodology. This means that registry use for trial pre-screening may be much more important than reported in this review. Furthermore, studies reporting use of population and disease registries for trial pre-screening have failed to address questions around data privacy and protection. The majority of disease registries around the world are accessible by two types of users: patients, who may directly fill out information into the registries, and health care professionals. These registries have data agreement in place, regarding privacy, sharing, and use such as data extraction for research purposes. When pre-screening for clinical trials, clinical trial sponsors do request pre-screening logs. This is done for financial reasons, as clinical research teams do negotiate in their clinical trial budget to be compensated for the time spent pre-screening patients for a specific trial. Pre-screening logs are provided by sponsors and collect limited data respecting information privacy regulations applying locally, such as General Data Protection Regulation (GDPR) in the European Union. It is essential to continue using tools such as pre-screening logs to serve as buffers to minimize data sharing from registries to sponsors (most often pharmaceutical companies) and maintain compliance with information privacy regulations. The main difference linked to this aspect would be observed between the USA and Europe, as the US regulation allows for race data to be collected which is not approved in Europe. This is limiting the evaluation of racial representativeness in European clinical trials, which may be biased by enrolling a vast majority of Caucasian participants.

Finally, as artificial intelligence (AI) is being developed, studies are now reporting use of machine learning for patient pre-screening into trials: Su et al. recently cited a pilot trial from the Mayo Clinic in Rochester using an AI-based trial matching system [2]. The paper reported an enrollment increase of 80% due to the quick and accurate patient matching to the oncology trial run at the Mayo Clinic [2], a system that could be applied to patient registries. Oncology has also brought us algorithms for clinical trial pre-screening, specifically Evolutionary Strategy algorithms (ES algorithms) [100, 101], that are commonly used in machine learning [102]. Ni et al. reported a 450% increase in efficacy of clinical trial pre-screening using electronic health record and not a patient registry, despite the fact that 10% of eligible patients were missed in the process [101]. More globally, data-driven technologies and strategies are more and more being reported in the literature, whether it is supporting prevention, diagnosis, or decision-making [103–106]. Such strategies’ impact on time optimization and associated cost reduction could be of great aid both to small trial centers working with limited staff and resources, and bigger trial centers dealing with a large volume of patients and trials.

Future studies are needed to address the limitations specific to certain disease fields to better describe the disease-specific needs around the use of registries for clinical trial pre-screening.

## Conclusion

In conclusion, we aimed to describe the type of registries used, disease areas, type of clinical trials linked to the registry-based pre-screening, and potential assets the method brought to the pre-screening process. Only 24 studies between 2014 and 2022 reported using population and disease registries for clinical trial pre-screening despite time optimization and financial advantages using the method. A majority of the registries used were on a national level, and half of the trials for which pre-screening was performed were drug trials. Pre-screening strategies remain under-reported, and the use of population and disease registries for trial pre-screening may be much more important than what is described in this review, both for drug trials and non-drug trials. Our review is therefore stressing the need for standardized methodological guidelines for clinical trial pre-screening and encourages reporting of pre-screening processes in trial protocols and publications.

### Supplementary Information

**Supplementary Material 1.**

## Data Availability

Data is accessible upon request (full search and excel master document supporting screening, exported from Rayyan).

## Abbreviations

ALS: Amyotrophic lateral sclerosis
VC: Vital capacity
NCT: National clinical trial
SOPs: Standard operating procedures
EHR: Electronic health record
ICTRP: International Clinical Trial Registry Platform
WHO: World Health Organization
MeSH: Medical subject heading
LISA-R: List of Included Studies and quality Assurance in Review
RoB: Risk of bias
PECO: Patient/Exposure/Comparator/Outcome
PICO: Patient/Intervention/Comparator/Outcome
MND: Motor neuron disease
AI: Artificial intelligence
ES: Evolutionary Strategy
