# Supplementary material for: The usage of population and disease registries as pre-screening tools for clinical trials, a systematic review
Source: Syst Rev. 2024 Apr 23;13:111. doi: 10.1186/s13643-024-02533-0 (PMC11040983; doi:10.1186/s13643-024-02533-0)
Supplement: Supplementary file 1 — Supplementary Material 1. [file 13643_2024_2533_MOESM1_ESM.docx]

**Title:** The usage of population and disease registries as pre-screening tools for clinical trials, a systematic review

**Authors:** Juliette Foucher^1,2^, Louisa Azizi^1^, Linn Öijerstedt^1,2^, Ulf Kläppe^1,2^, Caroline Ingre^1,2^

­­­­­­­­­­­­­­­­­^1^ Department of Clinical Neuroscience, Karolinska Institutet, Stockholm, Sweden

^2^ Department of Neurology, Karolinska University Hospital, Stockholm, Sweden

**Supplementary material**

**Supplementary material 1 – Search strategies for Medline (a), Embase (b) and Web of Science (c)**

a. Medline

| Interface: Ovid MEDLINE(R) ALL  Date of Search: 22 June 2023  Number of hits: 813  Comment: In Ovid, two or more words are automatically searched as phrases; i.e. no quotation marks are needed  The Ovid MEDLINE®️ database contains records with the following possible status besides MEDLINE: Publisher, In-Data-Review, In-Process and PubMed-not-MEDLINE records from NLM. | Field labels   - exp/ = exploded MeSH term - / = non exploded MeSH term - .ti,ab,kf. = title, abstract and author keywords - adjx = within x words, regardless of order - * = truncation of word for alternate endings |
| --- | --- |
| Database(s): **Ovid MEDLINE(R) ALL**1946 to June 15, 2023 Search Strategy:   \| **#** \| **Searches** \| **Results** \| \| --- \| --- \| --- \| \| 1 \| Registries/ \| 108273 \| \| 2 \| ((registry or registries or register?) not (clinical trial* regist* or Cochrane Central Regist*)).ti,ab,kf. \| 221348 \| \| 3 \| 1 or 2 \| 255176 \| \| 4 \| Patient selection/ \| 69405 \| \| 5 \| ((selection or recruit*) adj2 patient?).kf. \| 915 \| \| 6 \| ((selection or recruit*) and patient?).ti. \| 7987 \| \| 7 \| (pre-screen* or prescreen*).ti,ab,kf. \| 4385 \| \| 8 \| (((selection or recruit*) adj2 patient?) not trial registration).ab. \| 78895 \| \| 9 \| or/4-8 \| 150540 \| \| 10 \| exp Clinical trials as topic/ \| 382754 \| \| 11 \| (trial? or rct?).ti,ab,kf. \| 1318926 \| \| 12 \| 10 or 11 \| 1485122 \| \| 13 \| 3 and 9 and 12 \| 1507 \| \| 14 \| limit 13 to (english language and yr="2014 - 2022") \| 813 \| | |

b. Embase

| Interface: embase.com  Date of Search: 22 June 2023  Number of hits: 843  Comment: Emtree is the controlled vocabulary in Embase | Field labels   - /exp = exploded Emtree term - /de = non exploded Emtree term - ti,ab,kw = title, abstract and author keywords - NEAR/x = within x words, regardless of order - * = truncation of word for alternate endings |
| --- | --- |
| **No.**  **Query**  **Results**  **843**  **#17**  **#15** NOT **#16**  **564**  **#16**  **#3** AND **#10** AND **#13** AND [english]/lim AND [2014-2022]/py AND [conference abstract]/lim  **1,407**  **#15**  **#3** AND **#10** AND **#13** AND [english]/lim AND [2014-2022]/py  **2,258**  **#14**  **#3** AND **#10** AND **#13**  **2,054,589**  **#13**  **#11** OR **#12**  **1,884,815**  **#12**  trial$:ti,ab,kw OR rct$:ti,ab,kw  **431,917**  **#11**  **'clinical trial (topic)'**/exp  **229,906**  **#10**  **#8** OR **#9**  **139,237**  **#9**  (((**selection** OR **recruit***) NEAR/2 patient$):ab) NOT **'trial registration'**:ab  **122,455**  **#8**  **#4** OR **#5** OR **#6** OR **#7**  **6,535**  **#7**  **'pre screen*'**:ti,ab,kw OR **prescreen***:ti,ab,kw  **11,560**  **#6**  (**selection**:ti OR **recruit***:ti) AND patient$:ti  **1,614**  **#5**  ((**selection** OR **recruit***) NEAR/2 patient$):kw  **108,785**  **#4**  **'patient selection'**/exp  **397,364**  **#3**  **#1** OR **#2**  **358,389**  **#2**  (**registry**:ti,ab,kw OR **registries**:ti,ab,kw OR register$:ti,ab,kw) NOT (**'clinical trial* regist*'**:ti,ab,kw OR **'cochrane central regist*'**:ti,ab,kw)  **190,893**  **#1**  **'register'**/exp | |

c. Web of Science Core Collection

| Interface: Clarivate Analytics  Editions = A&HCI , ESCI , SCI-EXPANDED , SSCI  Date of Search: 22 June 2023  Number of hits: 713 | Field labels   - TS/Topic = title, abstract, author keywords and Keywords Plus - NEAR/x = within x words, regardless of order - * = truncation of word for alternate endings   Note: the *Exact search*-function was used for all the searches |
| --- | --- |
| \| # \| Search Query \| Results \| \| --- \| --- \| --- \| \| 1 \| TS=((registry OR registries OR register$ ) NOT ("clinical trial* regist*" OR "Cochrane Central Regist*" )) \| 275576 \| \| 2 \| AK=((selection OR recruit*) NEAR/2 patient$) \| 1558 \| \| 3 \| TI=((selection OR recruit* ) AND patient$ ) \| 10082 \| \| 4 \| TS=(pre-screen* OR prescreen* ) \| 5370 \| \| 5 \| AB=(((selection OR recruit* ) NEAR/2 patient$ ) NOT "trial registration" ) \| 80751 \| \| 6 \| #5 OR #4 OR #3 OR #2 \| 95266 \| \| 7 \| TS=(trial$ OR rct$ ) \| 1851447 \| \| 8 \| #7 AND #6 AND #1 \| 1100 \| \| 9 \| (#7 AND #6 AND #1) AND (PY==("2014" OR "2015" OR "2016" OR "2017" OR "2018" OR "2020" OR "2021" OR "2022" OR "2019") AND LA==("ENGLISH")) \| 713 \| | |

**Supplementary material 2 – Blank quality assurance tool to assess included studies reporting the use of a patient/disease registry for clinical trial pre-screening (LISA-R tool – List of Included Studies and quality Assurance in Review)**

| **Item** | *Yes= 1 / No= 0* |
| --- | --- |
| Was the registry's name provided? (Yes/No) |  |
| Was the consenting process into the registry described? (Yes/No) |  |
| Was demographic data coming from the registry included? (Yes/No) |  |
| Was there mention of a specific clinical trial for which the registry was used for pre-screening? (Yes/No) |  |
| Was a trial NCT number mentionned? (Yes/No) |  |
| Was the methodology of the registry's use for clinical trial pre-screening described? (Yes/No) |  |
| Did the paper report on the number of pre-screened pts from the registry got enrolled / could be enrolled in the clinical trial? (Yes/No) |  |
| Do the conclusions drawn in the research report flow from the analysis, or interpretation, of the data? (Yes/No) |  |
| Where Conflict of interest described? (Yes/No) |  |
| Was funding for this research described? (Yes/No) |  |
| Does the paper reports benefits of registry use for trial pre-screening? (Yes/No) |  |
| **Total Score** | Between 0 and 11 |
| **Assessment** | - Total > 8: high quality - 6 < Total < 8: moderate quality - Total < 6: low quality |

© Copyright, 2023. All Rights Reserved. The author being Juliette Foucher.

| **Supplementary material 3 – LISAR-R** | | | | | | | | | | | | | | |
| --- | --- | --- | --- | --- | --- | --- | --- | --- | --- | --- | --- | --- | --- | --- |
|  | **Question 1** | **Question 2** | **Question 3** | **Question 4** | **Question 5** | **Question 6** | **Question 7** | **Question 8** | **Question 9** | **Question 10** | **Question 11** | **Total Score** | **Q/A level (<6: low, between 6 and 8: moderate, >8:high)** | **Decision (low quality: exclude, moderate and high quality: include)** |
| **#** | Was the registry's name provided? | Was the consenting process into the registry described? | Was demographic data coming from the registry included? | Was there mention of a specific clinical trial for which the registry was used for pre-screening? | Was a trial NCT number mentionned? | Was the methodology of the registry's use for clinical trial pre-screening described? | Did the paper report on the number of pre-screened pts from the registry got enrolled / could be enrolled in the clinical trial? | Do the conclusions drawn in the research report flow from the analysis, or interpretation, of the data? | Were Conflict of interests described? | Was funding for this research described? | Does the paper reports benefits of registry use for trial pre-screening? |  |  |  |
| 1 | Yes | No | Yes | No | No | Yes | No | Yes | Yes | Yes | Yes | 7 | Moderate | Include |
| 2 | Yes | No | Yes | No | No | Yes | No | Yes | Yes | Yes | Yes | 7 | Moderate | Include |
| 3 | Yes | No | Yes | Yes | Yes | Yes | Yes | Yes | Yes | Yes | Yes | 10 | High | Include |
| 4 | Yes | Yes | No | Yes | No | No | No | No | Yes | Yes | No | 5 | Low | Exclude |
| 5 | Yes | Yes | Yes | Yes | No | Yes | Yes | Yes | Yes | Yes | Yes | 10 | High | Include |
| 6 | No | No | Yes | Yes | Yes | Yes | No | Yes | Yes | Yes | No | 7 | Moderate | Include |
| 7 | Yes | No | Yes | Yes | No | Yes | Yes | Yes | Yes | No | Yes | 8 | Moderate | Include |
| 8 | Yes | No | Yes | No | No | Yes | Yes | Yes | Yes | Yes | Yes | 8 | Moderate | Include |
| 9 | Yes | No | Yes | No | No | Yes | Yes | Yes | Yes | Yes | Yes | 8 | Moderate | Include |
| 10 | Yes | No | Yes | Yes | No | No | Yes | Yes | Yes | Yes | No | 7 | Moderate | Include |
| 11 | Yes | Yes | Yes | Yes | Yes | Yes | Yes | Yes | Yes | Yes | Yes | 11 | High | Include |
| 12 | Yes | No | Yes | Yes | Yes | No | Yes | Yes | Yes | No | No | 7 | Moderate | Include |
| 13 | Yes | No | Yes | Yes | No | No | Yes | Yes | Yes | Yes | Yes | 8 | Moderate | Include |
| 14 | Yes | Yes | Yes | Yes | Yes | Yes | Yes | Yes | Yes | Yes | Yes | 11 | High | Include |
| 15 | Yes | No | Yes | No | No | Yes | Yes | Yes | Yes | Yes | No | 7 | Moderate | Include |
| 16 | Yes | No | Yes | Yes | Yes | Yes | Yes | Yes | Yes | Yes | No | 9 | High | Include |
| 17 | Yes | No | Yes | Yes | Yes | No | Yes | Yes | Yes | Yes | No | 8 | Moderate | Include |
| 18 | Yes | No | Yes | Yes | Yes | Yes | Yes | Yes | Yes | Yes | No | 9 | High | Include |
| 19 | Yes | No | Yes | Yes | Yes | Yes | Yes | Yes | Yes | Yes | Yes | 10 | High | Include |
| 20 | No | No | No | Yes | Yes | Yes | Yes | Yes | No | Yes | Yes | 7 | Moderate | Include |
| 21 | Yes | No | Yes | Yes | Yes | Yes | Yes | Yes | Yes | Yes | Yes | 10 | High | Include |
| 22 | Yes | No | Yes | Yes | Yes | No | Yes | Yes | Yes | Yes | No | 8 | Moderate | Include |
| 23 | Yes | No | Yes | Yes | No | No | Yes | Yes | Yes | Yes | No | 7 | Moderate | Include |
| 24 | Yes | Yes | No | Yes | No | Yes | Yes | Yes | Yes | Yes | Yes | 9 | High | Include |
| 25 | Yes | No | Yes | Yes | No | Yes | Yes | Yes | Yes | Yes | No | 8 | Moderate | Include |

**Supplementary material 3.a – List of included studies and quality assurance in review tool (LISA-R)**

**Supplementary material 3.b – Detailed reference used with the List of included studies and quality assurance in review tool (LISA-R)**

| **#** | **Authors** | **Title** | **Year** | **Journal** | **DOI** | **LISA-R Decision** |
| --- | --- | --- | --- | --- | --- | --- |
| 1 | Xiang *et al.* | Brief Report: Implementation of a Universal Prescreening Protocol to Increase Recruitment to Lung Cancer Studies at a Veterans Affairs Cancer Center | 2022 | JTO Clinical and Research Reports | 10.1016/j.jtocrr.2022.100357 | Included |
| 2 | Wu *et al.* | Prescreening to Increase Therapeutic Oncology Trial Enrollment at the Largest Public Hospital in the United States | 2022 | JCO Oncology Practice | 10.1200/OP.21.00629 | Included |
| 3 | Valle *et al.* | Recruitment of young adult cancer survivors into a randomized controlled trial of an mHealth physical activity intervention | 2022 | Trials [Electronic Resource] | 10.1186/s13063-022-06148-5 | Included |
| 4 | Farey *et al.* | Dual mobility versus conventional total hip arthroplasty in femoral neck fractures (DISTINCT): protocol for a registry-nested, open-label, cluster-randomised crossover trial | 2022 | BMJ Open | 10.1136/bmjopen-2022-064478 | Excluded |
| 5 | Danila *et al.* | Evaluation of an Intervention to Support Patient-Rheumatologist Conversations About Escalating Treatment in Patients with Rheumatoid Arthritis: A Proof-of-Principle Study | 2022 | ACR open rheumatology | 10.1002/acr2.11393 | Included |
| 6 | Shadyab *et al.* | Recruitment of a multi-site randomized controlled trial of aerobic exercise for older adults with amnestic mild cognitive impairment: The EXERT trial | 2021 | Alzheimer's & Dementia | 10.1002/alz.12401 | Included |
| 7 | Mehta *et al.* | Recruitment of Patients With Amyotrophic Lateral Sclerosis for Clinical Trials and Epidemiological Studies: Descriptive Study of the National ALS Registry's Research Notification Mechanism | 2021 | Journal of Medical Internet Research | 10.2196/28021 | Included |
| 8 | Guerra *et al.* | Pancreatic Cancer Clinical Treatment Trials Accrual: A Closer Look at Participation Rates | 2021 | American Journal of Clinical Oncology | 10.1097/COC.0000000000000807 | Included |
| 9 | Green *et al.* | Implementation of a molecular tumor registry to support the adoption of precision oncology within an Academic Medical Center: The Duke University Experience | 2021 | JCO Precision Oncology | 10.1200/PO.21.00030 | Included |
| 10 | van der Hout *et al.* | Role of eHealth application Oncokompas in supporting self-management of symptoms and health-related quality of life in cancer survivors: a randomised, controlled trial | 2020 | Lancet Oncology | 10.1016/S1470-2045(19)30675-8 | Included |
| 11 | Huebner *et al.* | Heregulin (HRG) assessment for clinical trial eligibility testing in a molecular registry (PRAEGNANT) in Germany | 2020 | BMC Cancer | 10.1186/s12885-020-07546-1 | Included |
| 12 | Toth *et al.* | Validation of the all-comers design: Results of the TARGET-AC substudy | 2020 | American Heart Journal | 10.1016/j.ahj.2019.10.019 | Included |
| 13 | Lasch *et al.* | Commentary: On the levels of patient selection in registry-based randomized controlled trials | 2019 | Trials | 10.1186/s13063-019-3214-x | Included |
| 14 | De Antonio *et al.* | The DM-scope registry: a rare disease innovative framework bridging the gap between research and medical care | 2019 | Orphanet Journal Of Rare Diseases | 10.1186/s13023-019-1088-3 | Included |
| 15 | Tamborlane *et al.* | Eligibility for clinical trials is limited for youth with type 2 diabetes: Insights from the Pediatric Diabetes Consortium T2D Clinic Registry | 2018 | Pediatric Diabetes | 10.1111/pedi.12763 | Included |
| 16 | Darmon *et al.* | External applicability of the COMPASS trial: an analysis of the reduction of atherothrombosis for continued health (REACH) registry | 2018 | European Heart Journal | 10.1093/eurheartj/ehx658 | Included |
| 17 | Oni *et al.* | Eligibility for clinical trials in primary Sjogren's syndrome: lessons from the UK Primary Sjogren's Syndrome Registry | 2016 | Rheumatology | 10.1093/rheumatology/kev373 | Included |
| 18 | Brown *et al.* | A randomized phase II dose-response exercise trial among colon cancer survivors: Purpose, study design, methods, and recruitment results | 2016 | Contemporary Clinical Trials | 10.1016/j.cct.2016.03.001 | Included |
| 19 | Aung *et al.* | Cost-effective recruitment methods for a large randomised trial in people with diabetes: A Study of Cardiovascular Events iN Diabetes (ASCEND) | 2016 | Trials | 10.1186/s13063-016-1354-9 | Included |
| 20 | Russo *et al.* | Chronic obstructive pulmonary disease self-management activation research trial (COPD-SMART): results of recruitment and baseline patient characteristics | 2015 | Contemporary Clinical Trials | 10.1016/j.cct.2015.01.018 | Included |
| 21 | Heywood *et al.* | Effective recruitment of participants to a phase I study using the internet and publicity releases through charities and patient organisations: analysis of the adaptive study of IL-2 dose on regulatory T cells in type 1 diabetes (DILT1D) | 2015 | Trials | 10.1186/s13063-015-0583-7 | Included |
| 22 | Heidrich *et al.* | Treatment extension of pegylated interferon alpha and ribavirin does not improve SVR in patients with genotypes 2/3 without rapid virological response (OPTEX Trial): A prospective, randomized, two-arm, multicentre phase IV clinical trial | 2015 | PLoS ONE | 10.1371/journal.pone.0128069 | Included |
| 23 | Achiron *et al.* | Effect of Alfacalcidol on multiple sclerosis-related fatigue: A randomized, double-blind placebo-controlled study | 2015 | Multiple Sclerosis | 10.1177/1352458514554053 | Included |
| 24 | Curtis *et al.* | Use of health plan combined with registry data to predict clinical trial recruitment | 2014 | Clinical Trials | 10.1177/1740774513512185 | Included |
| 25 | Ashing *et al.* | A telephonic-based trial to reduce depressive symptoms among Latina breast cancer survivors | 2014 | Psycho-Oncology | 10.1002/pon.3441 | Included |

**Supplementary material 4 - Included papers, descriptive characteristics**

| **Table 2 – Included papers, descriptive characteristics** | | | | | | | | |
| --- | --- | --- | --- | --- | --- | --- | --- | --- |
| **#** | **Authors** | **Simplified / Authors** | **Title** | **Year** | **Journal** | **Registry Country** | **Disease domain** | **Objectives of the article (citations)** |
| **1** | Xiang, J. J. and Roy, A. and Summers, C. and Delvy, M. and O'Donovan, J. and Christensen, J. and Dwy, C. and Perry, L. and Connery, D. and Rose, M. G. and Sheehan, K. and Chao, H. H. | Xiang et al. | Brief Report: Implementation of a Universal Prescreening Protocol to Increase Recruitment to Lung Cancer Studies at a Veterans Affairs Cancer Center | 2022 | JTO Clinical and Research Reports | US | Oncology | Describe the real-world experience of implementing a standardized, universal prescreening protocol and its impact  on research recruitment to thoracic oncology studies |
|  |  |  |  |  |  |  |  |  |
|  |  |  |  |  |  |  |  |  |
| **2** | Wu, J. and Yakubov, A. and Abdul-Hay, M. and Love, E. and Kroening, G. and Cohen, D. and Spalink, C. and Joshi, A. and Balar, A. and Joseph, K. A. and Ravenell, J. and Mehnert, J. | Wu et al. | Prescreening to Increase Therapeutic Oncology Trial Enrollment at the Largest Public Hospital in the United States | 2022 | JCO Oncology Practice | US | Oncology | Implemented a quality improvement program to increase therapeutic trial enrollment |
| **3** | Valle, C. G. and Camp, L. N. and Diamond, M. and Nezami, B. T. and LaRose, J. G. and Pinto, B. M. and Tate, D. F. | Valle et al. | Recruitment of young adult cancer survivors into a randomized controlled trial of an mHealth physical activity intervention | 2022 | Trials [Electronic Resource] | US | Oncology | Describes recruitment strategies used in the IMproving Physical Activityafter Cancer Treatment (IMPACT) study |
| **4** | Danila, M. I. and Chen, L. and Ruderman, E. M. and Owensby, J. K. and O'Beirne, R. and Melnick, J. A. and Harrold, L. R. and Curtis, D. and Nowell, W. B. and Curtis, J. R. | Danila et al. | Evaluation of an Intervention to Support Patient-Rheumatologist Conversations About Escalating Treatment in Patients with Rheumatoid Arthritis: A Proof-of-Principle Study | 2022 | ACR OPEN RHEUMATOLOGY | US | Inflammatory | Test whether an online video intervention discussing appropriate treatment escalation improves willingness to change treatment in people living with rheumatoid arthritis |
| **5** | Shadyab, A. H. and LaCroix, A. Z. and Feldman, H. H. and van Dyck, C. H. and Okonkwo, O. C. and Tam, S. P. and Fairchild, J. K. and Welsh-Bohmer, K. A. and Matthews, G. and Bennett, D. and Shadyab, A. A. and Schafer, K. A. and Morrison, R. H. and Kipperman, S. A. and Mason, J. and Tan, D. and Thomas, R. G. and Cotman, C. W. and Baker, L. D. | Shadyab et al. | Recruitment of a multi-site randomized controlled trial of aerobic exercise for older adults with amnestic mild cognitive impairment: The EXERT trial | 2021 | Alzheimer's & Dementia | US | Neurology | Describe the successes and challenges we encountered in recruiting adults with MCI into the EXERT trial |
| **6** | Mehta, P. and Raymond, J. and Han, M. K. and Larson, T. and Berry, J. D. and Paganoni, S. and Mitsumoto, H. and Bedlack, R. S. and Horton, D. K. | Mehta et al. | Recruitment of Patients With Amyotrophic Lateral Sclerosis for Clinical Trials and Epidemiological Studies: Descriptive Study of the National ALS Registry's Research Notification Mechanism | 2021 | Journal of Medical Internet Research | US | Neurology / Neuromuscular | Describe how the federal Agency for Toxic Substances and Disease Registry’s (ATSDR) National ALS Registry is linking PALS to scientists who are conducting research, clinical trials, and epidemiological studies |
| **7** | Guerra, C. E. and Kelly, S. and Redlinger, C. and Hernandez, P. and Glanz, K. | Guerra et al. | Pancreatic Cancer Clinical Treatment Trials Accrual: A Closer Look at Participation Rates | 2021 | American Journal of Clinical Oncology | US | Oncology | To calculate participation rates based on eligibility for and enrollment in pancreatic cancer clinical trials |
| **8** | Green, M. F. and Bell, J. L. and Hubbard, C. B. and McCall, S. J. and McKinney, M. S. and Riedel, J. E. and Menendez, C. S. and Abbruzzese, J. L. and Strickler, J. H. and Datto, M. B. | Green et al. | Implementation of a molecular tumor registry to support the adoption of precision oncology within an Academic Medical Center: The Duke University Experience | 2021 | JCO Precision Oncology | US | Oncology | Present an informatic system to organize and store molecular data and make it available for timely physician support |
| **9** | van der Hout, A. and van Uden-Kraan, C. F. and Holtmaat, K. and Jansen, F. and Lissenberg-Witte, B. I. and Nieuwenhuijzen, G. A. P. and Hardillo, J. A. and Baatenburg de Jong, R. J. and Tiren-Verbeet, N. L. and Sommeijer, D. W. and de Heer, K. and Schaar, C. G. and Sedee, R. E. and Bosscha, K. and van den Brekel, M. W. M. and Petersen, J. F. and Westerman, M. and Honings, J. and Takes, R. P. and Houtenbos, I. and van den Broek, W. T. and de Bree, R. and Jansen, P. and Eerenstein, S. E. J. and Leemans, C. R. and Zijlstra, J. M. and Cuijpers, P. and van de Poll-Franse, L. V. and Verdonck-de Leeuw, I. M. | van de Hout et al. | Role of eHealth application Oncokompas in supporting self-management of symptoms and health-related quality of life in cancer survivors: a randomised, controlled trial | 2020 | Lancet Oncology | Netherlands | Oncology | Assess the efficacy, reach, and usage of Oncokompas, a web-based eHealth application that supports survivors in self-management by monitoring healthrelated quality of life (HRQOL) and cancer-generic and tumour-specific symptoms and obtaining tailored feedback with a personalised overview of supportive care options |
| **10** | Huebner, H. and Kurbacher, C. M. and Kuesters, G. and Hartkopf, A. D. and Lux, M. P. and Huober, J. and Volz, B. and Taran, F. A. and Overkamp, F. and Tesch, H. and Haberle, L. and Luftner, D. and Wallwiener, M. and Muller, V. and Beckmann, M. W. and Belleville, E. and Ruebner, M. and Untch, M. and Fasching, P. A. and Janni, W. and Fehm, T. N. and Kolberg, H. C. and Wallwiener, D. and Brucker, S. Y. and Schneeweiss, A. and Ettl, J. | Huebner et al. | Heregulin (HRG) assessment for clinical trial eligibility testing in a molecular registry (PRAEGNANT) in Germany | 2020 | BMC Cancer | Germany | Oncology | Investigate eligibility testing for the SHERBOC trial using the German PRAEGNANT registry |
| **11** | G, G. Toth. and Lansky, A. and Baumbach, A. and Kelbaek, H. and van Royen, N. and Holmvang, L. and Janssens, L. and Brugaletta, S. and Barbato, E. and Maillard, L. and Kiemeneij, F. and Naber, C. K. and Pucher, F. and Laursen, P. N. and Ameloot, K. and Robles, C. and Milkas, A. and Sevilla, J. and Jensen, C. and Wijns, W. | Toth et al. | Validation of the all-comers design: Results of the TARGET-AC substudy | 2020 | American Heart Journal | International | Cardiovascular | Evaluate how far an all-comers design in the context of clinical research can ensure the representation of the true all comers population |
| **12** | Lasch, F. and Weber, K. and Koch, A. | Lasch et al. | Commentary: On the levels of patient selection in registry-based randomized controlled trials | 2019 | Trials [Electronic Resource] | Sweden | Cardiovascular | To study the different levels of patient selection in rRCTs to achieve a better understanding of the possibilities, limitations, and the generalizability of rRCTs results. |
| **13** | De Antonio, M. and Dogan, C. and Daidj, F. and Eymard, B. and Puymirat, J. and Mathieu, J. and Gagnon, C. and Katsahian, S. and Hamroun, D. and Bassez, G. | De Antonio et al. | The DM-scope registry: a rare disease innovative framework bridging the gap between research and medical care | 2019 | Orphanet Journal Of Rare Diseases | France | Neurology / Neuromuscular | Describe the innovative concept of the DM-Scope Registry |
| **14** | Tamborlane, W. V. and Chang, P. and Kollman, C. and Klingensmith, G. J. and Ruedy, K. and Gal, R. L. and Van Name, M. and Bacha, F. and Willi, S. and Beck, R. W. | Tamborlane et al. | Eligibility for clinical trials is limited for youth with type 2 diabetes: Insights from the Pediatric Diabetes Consortium T2D Clinic Registry | 2018 | Pediatric Diabetes | US | Metabolic | To estimate the percentage of patients who would be excluded from current T2D trials based on out-of-range HbA1c levels based on PDC registry |
| **15** | Darmon, A. and Bhatt, D. L. and Elbez, Y. and Aboyans, V. and Anand, S. and Bosch, J. and Branch, K. R. and Connolly, S. J. and Dyal, L. and Eikelboom, J. W. and Fox, K. A. A. and Keltai, K. and Probstfield, J. and Yusuf, S. and Abtan, J. and Sorbets, E. and Eagle, K. A. and Ducrocq, G. and Steg, P. G. | Darmon et al. | External applicability of the COMPASS trial: an analysis of the reduction of atherothrombosis for continued health (REACH) registry | 2018 | European Heart Journal | International | Cardiovascular | Describe the proportion of patients eligible for the COMPASS trial within the Reduction of Atherothrombosis for Continued Health (REACH) registry, the reasons for ineligibility, and to put in perspective the characteristics and outcomes of trial-eligible patients from the REACH registry compared with those of patients enrolled in the reference aspirin arm of the COMPASS trial |
| **16** | Oni, C. and Mitchell, S. and James, K. and Ng, W. F. and Griffiths, B. and Hindmarsh, V. and Price, E. and Pease, C. T. and Emery, P. and Lanyon, P. and Jones, A. and Bombardieri, M. and Sutcliffe, N. and Pitzalis, C. and Hunter, J. and Gupta, M. and McLaren, J. and Cooper, A. and Regan, M. and Giles, I. and Isenberg, D. and Saravanan, V. and Coady, D. and Dasgupta, B. and McHugh, N. and Young-Min, S. and Moots, R. and Gendi, N. and Akil, M. and Barone, F. and Fisher, B. and Rauz, S. and Richards, A. and Bowman, S. J. | Oni et al. | Eligibility for clinical trials in primary Sjögren's syndrome: lessons from the UK Primary Sjogren's Syndrome Registry | 2016 | Rheumatology | UK | Autoimmune | Identify numbers of participants in the UK Primary Sjögren’s Syndrome Registry (UKPSSR) who would fulfil eligibility criteria for previous/current or potential clinical trials in primary SS |
| **17** | Brown, J. C. and Troxel, A. B. and Ky, B. and Damjanov, N. and Zemel, B. S. and Rickels, M. R. and Rhim, A. D. and Rustgi, A. K. and Courneya, K. S. and Schmitz, K. H. | Brown et al. | A randomized phase II dose-response exercise trial among colon cancer survivors: Purpose, study design, methods, and recruitment results | 2016 | Contemporary Clinical Trials | US | Oncology | Present recruitment results to describe what demographic, clinical, or geographic characteristics are associated with inquiry about study participation and randomization onto the study protocol |
| **18** | Aung, T. and Haynes, R. and Barton, J. and Cox, J. and Murawska, A. and Murphy, K. and Lay, M. and Armitage, J. and Bowman, L. | Aung et al. | Cost-effective recruitment methods for a large randomised trial in people with diabetes: A Study of Cardiovascular Events iN Diabetes (ASCEND) | 2016 | Trials [Electronic Resource] | UK | Endocrinology | Describe recruitment methods, study paticipation rate of patients with diabetes eligible for ASCEND trial |
| **19** | Russo, R. and Coultas, D. and Ashmore, J. and Peoples, J. and Sloan, J. and Jackson, B. E. and Uhm, M. and Singh, K. P. and Blair, S. N. and Bae, S. | Russo et al. | Chronic obstructive pulmonary disease self-management activation research trial (COPD-SMART): results of recruitment and baseline patient characteristics | 2015 | Contemporary Clinical Trials | US | Pulmonary | Describe the recruitment methods, study participation rate, and baseline characteristics of a representative sample of outpatients with COPD eligible for pulmonary rehabilitation participating in a trial |
| **20** | Heywood, J. and Evangelou, M. and Goymer, D. and Kennet, J. and Anselmiova, K. and Guy, C. and O'Brien, C. and Nutland, S. and Brown, J. and Walker, N. M. and Todd, J. A. and Waldron-Lynch, F. | Heywood et al. | Effective recruitment of participants to a phase I study using the internet and publicity releases through charities and patient organisations: analysis of the adaptive study of IL-2 dose on regulatory T cells in type 1 diabetes (DILT1D) | 2015 | Trials [Electronic Resource] | UK | Endocrinology | Descrube recruitment methods to the phase 1 trial DILT1D |
| **21** | Heidrich, B. and Cordes, H. J. and Klinker, H. and MÃller, B. and Naumann, U. and RÃssle, M. and Kraus, M. R. and BÃ¶ker, K. H. and Roggel, C. and Schuchmann, M. and Stoehr, A. and Trein, A. and Hardtke, S. and Gonnermann, A. and Koch, A. and Wedemeyer, H. and Manns, M. P. and Cornberg, M. | Heidrich et al. | Treatment extension of pegylated interferon alpha and ribavirin does not improve SVR in patients with genotypes 2/3 without rapid virological response (OPTEX Trial): A prospective, randomized, two-arm, multicentre phase IV clinical trial | 2015 | PLoS ONE | Germany | Hepatology | Assess the effects of prolonged treatment for HCV positive patients |
| **22** | Achiron, A. and Givon, U. and Magalashvili, D. and Dolev, M. and Liraz Zaltzman, S. and Kalron, A. and Stern, Y. and Mazor, Z. and Ladkani, D. and Barak, Y. | Achiron et al. | Effect of Alfacalcidol on multiple sclerosis-related fatigue: A randomized, double-blind placebo-controlled study | 2015 | Multiple Sclerosis | Israel | Neurology | Asses the use of Alfacadicol to treat fatigue amonst MS patients |
| **23** | Curtis, J. R. and Wright, N. C. and Xie, F. and Chen, L. and Zhang, J. and Saag, K. G. and Bharat, A. and Kremer, J. and Cofield, S. and Winthrop, K. and Delzell, E. | Curtis et al. | Use of health plan combined with registry data to predict clinical trial recruitment | 2014 | Clinical Trials | US | Inflammatory | Assess the use of health plan and registry for trial recruitment |
| **24** | Ashing, K. and Rosales, M. | Ashing et al. | A telephonic-based trial to reduce depressive symptoms among Latina breast cancer survivors | 2014 | Psycho-Oncology | US | Oncology | Asses the effects of internvation on breast cancer survivors depressive symptom |
